# Diversity Among American Dermatological Association Members by Sex and Geographic Region

**DOI:** 10.2196/47802

**Published:** 2024-01-10

**Authors:** Ramiro Rodriguez, Lachlan Anderson, Emily Woolhiser, Timothy Balmorez, Bailey Cook, Megan Hauptman, Jessica Kirk, Noah Keime, Robert P Dellavalle

**Affiliations:** 1 Department of Dermatology University of Colorado School of Medicine Aurora, CO United States; 2 Boston College Boston, MA United States; 3 College of Osteopathic Medicine Kansas City University Kansas, KS United States; 4 College of Osteopathic Medicine Touro University California Vallejo, CA United States; 5 Rocky Vista University College of Osteopathic Medicine Parker, CO United States; 6 College of Medicine University of Arizona Tucson, AZ United States; 7 Dermatology Service US Department of Veterans Affairs Rocky Mountain Regional Medical Center Aurora, CO United States; 8 Department of Epidemiology Colorado School of Public Health University of Colorado Anschutz Medical Campus Aurora, CO United States

**Keywords:** American Dermatological Association, disparity, representation, dermatology, urban, rural, dermatological society, diversity, inclusion, equity, sex, membership, acquisition, demographic

## Introduction

Professional societies create networking, mentorship, and research collaboration opportunities, but disparities in gender, sex, geographic, ethnic, and racial composition within societies disadvantage professional development among underrepresented individuals. Our group evaluated the American Dermatological Association (ADA) since election occurs through a nomination by existing members; we hypothesize this process creates gaps in representation. Given the professional implications for underrepresented individuals, this review aims to quantify the disparities in sex and geographic location of ADA members. Ethnicity/race was not analyzed because the information was not publicly available.

## Methods

### Overview

In February 2023, the ADA directory identified 767 members. Two independent reviewers recorded member names, self-identified sex, city, and state listed on their national practitioner identifier, and those who were deceased; a third reviewer resolved data conflicts. Sex was identified on national practitioner identifier databases. Data were omitted for retired, deceased, or unidentified members. The statistical analysis was performed using R software (R Foundation for Statistical Computing), and the package “usmap” was used to create the figure. The directory was updated to include the 2023 inductees.

### Ethical Considerations

Data was publicly available and deidentified, and did not require institutional review board review.

## Results

**Figure 1:**
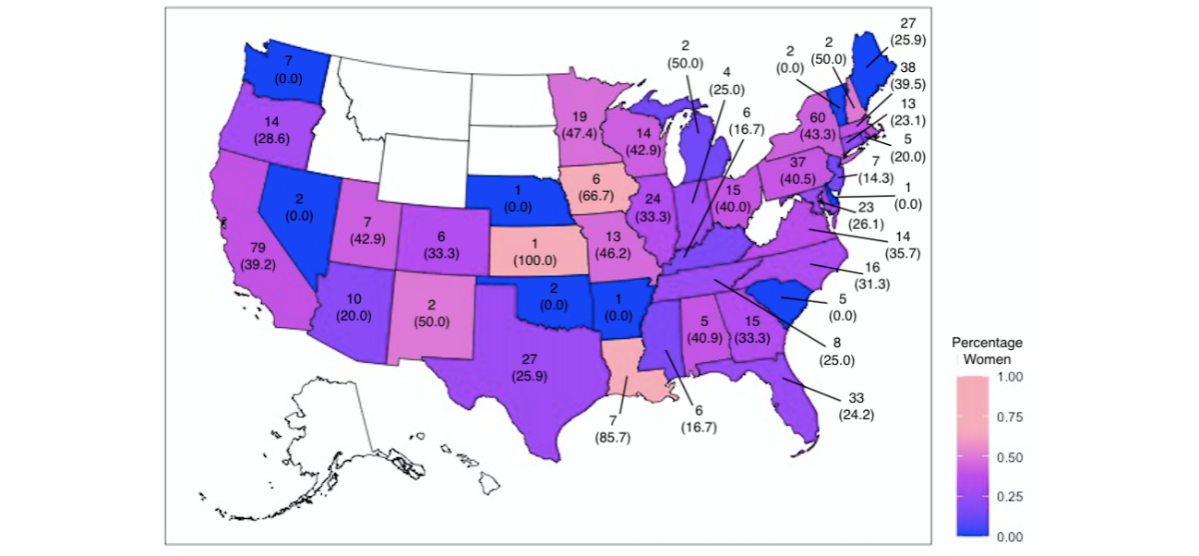
Representation of the American Dermatological Association members by sex and geographic region.

Of the 688 ADA members, 227 (33%) were female and 461 (67%) were male. A total of 581 (84.4%) members practiced in the United States, while 107 (15.6%) members practiced internationally; 26 (24.3%) of the 107 international members were female, and 81 (75.7%) international members were male. Among the 41 represented states, 2 had a similar number of male and female members (Figure 1). The top 5 states represented 247 (42.5%) members: California had 79 (13.6%) members, followed by 60 (10.3%) members in New York, 38 (6.5%) members in Massachusetts, 37 (6.3%) members in Pennsylvania, and 33 (5.6%) members in Florida (Table 1).

**Table 1:** Breakdown of American Dermatological Association membership by US region and top 10 states in female membership.

US regions	Members, n (%)	Female members, n (%)	Members per 1,000,000 people, n	Female members per 1,000,000 people, n
Northeast	166 (28.6)	62 (37.3)	2.90	1.08
South	176 (30.3)	53 (30.1)	1.38	0.42
Midwest	112 (19.3)	43 (38.4)	1.63	0.62
West	127 (21.9)	43 (33.9)	1.61	0.55
Total	581 (100.0)	201 (34.6)	1.75	0.61

## Discussion

Our study demonstrates that ADA membership does not represent the female dermatology workforce relative to geographic location and academic practice setting. Per Centers for Medicare & Medicaid Services data from 2020 and dividing US regions per the US Census Bureau criteria, female dermatologists ranged from 1430 of 1508 (48.7%) to 1148 of 1043 (52.4%) of the workforce in all regions of the United States [1]. In academic dermatology, the female workforce increased from 18 of 167 (10.8%) in 1970 to 749 of 1464 (51.2%) in 2018 [2]. Furthermore, as of 2020, 1125 (47.6%) of 2363 dermatologists who graduated medical school 28-36 years ago after graduating medical school are female [1], suggesting a diversified candidate pool for late-career recognitions like ADA membership.

Societies should aim to represent the dermatology workforce, which by extension should aim to represent the diverse composition of the United States. Data demonstrates direct benefits to patients stemming from a diverse workforce. For instance, an analysis of practice characteristics using the Black Dermatologist Directory identified 221 individuals (80% female). It was found that Black dermatologists served a higher proportion of non-Hispanic Black patients relative to other dermatologists (21.0 vs 2.7; *P*<.001) [3]. This data suggests a racial concordance preference, which can impact patient outcomes. For instance, data shows an 11% decrease in primary medication nonadherence among racial concordant Black dermatologists–Black patient dyads, independent of insurance status [4]. Research on ethnic/racial concordance can differ between ethnic/racial groups; however, cultural sensitivity is cited as a component of positive interactions [5]. Thus, honoring underrepresented individuals and diversifying professional societies can encourage cultural sensitivity among dermatologists through interactions with each other.

For dermatology-specific professional societies, data quantifying the impact of increased female representation is limited. However, interviews [6] of a women-focused professional organization report improved academic advancement, leadership experiences, awards, promotions, mentorship, and peer support, and reduced professional isolation. Other themes were the development of initiatives addressing systemic gender inequities/challenges like navigating bias, promoting pay equity, and family-friendly workplace policies. Given these benefits, there is a clear need for improved female representation in professional societies.

Specifically for the ADA, per the bylaws [7], candidates undergo membership proposition, review, and evaluation by a membership committee before proceeding to a ballot election. ADA leadership can promote diversity in different steps. For example, societies like the American Academy of Dermatology and The Skin of Color Society have mentorship programs dedicated to increasing diversity. A similar program may help identify competitive individuals for ADA membership to help improve their recognition among ADA members. In addition, including a race/sex-conscious nomination round can help diversify the pool of candidate reviews. Limitations of this study include the moment-in-time design and the exclusion of the race/ethnicity of members; the data needed to address these points could show important trends that demonstrate increased diversity. Future research can focus on evaluating the epidemiological characteristics of membership within other dermatologic societies, how these societies have changed over time, and identifying outcome measures to quantify the impact that diverse professional societies have on professional development.

## Abbreviations

ADA: American Dermatological Association
